# Functionalized-CNT Polymer Composite for Microwave and Electromagnetic Shielding

**DOI:** 10.3390/polym13223907

**Published:** 2021-11-12

**Authors:** Muhammed Kallumottakkal, Mousa I. Hussein, Yousef Haik, Tarik Bin Abdul Latef

**Affiliations:** 1 Department of Electrical Engineering, United Arab Emirates University, Al Ain 15551, United Arab Emirates; 201770122@uaeu.ac.ae; 2 Department of Mechanical and Industrial Engineering, Texas A & M University-Kingsville, Kingsville, TX 78363, USA; Yousef.Haik@tamuk.edu; 3 Electrical Engineering Department, University of Malaya, Kuala Lumpur 50603, Malaysia; tariqlatef@um.edu.my

**Keywords:** functionalized CNT, microwave absorption, dielectric, reflection loss

## Abstract

In this research work, we studied the microwave properties of multi-wall carbon nanotube (MWCNT) surface functionalized with metallic oxides composites. Three different concentrations (5%, 10%, and 20%) of metallic oxides were used, namely cobalt, iron, and cobalt ferrite. The surface-decorated CNTS were impregnated into polyurethane (PU) matrix. The surface-decorated MWCNTs and the MWCNTs-PU composites were characterized using electron microscopy. The dielectric properties of the samples are studied using an open-ended coaxial probe technique in a wide frequency range of (5–50 GHz). The metallic oxide-decorated surface MWCNTs-PU composites demonstrated different microwave-frequency absorption characteristics depending on the concentration of the metallic oxides.

## 1. Introduction

Microwave absorbing materials (MAMs) have become an essential part of the stealth defensive system for all military platforms [1,2] and communication and information processing technologies [3] due to the growing environmental emissions of microwave irradiation in our surroundings. Advances in material science and engineering have resulted in the creation of composite materials with high microwave absorption capacities while keeping the weight constraint in mind, which is critical in aeronautical applications. By Impeding absorbing charges (magnetic or dielectric) into a host matrix material creates this type of composite materials [4]. Microwave absorbers are commonly categorized as dielectric or magnetic absorbing materials in order to evaluate microwave absorption, dielectric absorbers depending on electronic polarization, ion polarization, and intrinsic electrical dipolar polarization in their materials and magnetic material on the magnetic properties [5]. The magnetic material composite can provide high absorption but it has high density [6]. Due to dielectric and magnetic losses, magnetic charges based on ferromagnetic metals such as iron, nickel, cobalt, or their alloys are used for permitting significant absorption in various spectral regions of interest [7]. The lightweight magnetic absorbers are much needed to nullify the effects of microwave radiation and it has a wide variety of application.

Recently, carbon allotropes such as graphene, MXenes, carbon nanotubes (CNTs), and carbon fiber have attracted increasing attention owing to their EMI shielding characteristics and being lightweight. Moreover, nanocomposites of CNT (carbon nanotubes) and magnetic metallic materials have attracted significant attention and much work has been done to manufacture and investigate their properties [8]. Cylindrical-shaped carbon nanotubes (CNT) consisting of either single-wall CNT (SWCNT) or multi-wall CNT (MWCNT) are well-known as wideband absorbing materials among different allotropic types of carbon atoms. These materials have strong mechanical and thermal properties and are lightweight [9,10]. Utilizing the lightweight of CNT and high absorption efficiency of magnetic material lead to the development of functionalized composites. Functionalization can be done either by filling or coating on the CNT, allowing to tune the absorption properties [11]. The polyurethane-based CNT composites are recognized among various magnetic absorbers for their outstanding mechanical properties [12]. The main contribution to absorption comes from magnetic (μ″) and dielectric (ε″) losses [6]. The majority of published studies concern composites in which carbon nanotubes are disseminated in an epoxy resin matrix, or to a lesser degree, in polyethylene, wax, or paraffin host media. Due to its excellent mechanical properties such as flexibility, resistance to bending, and ease of production in large quantities polyurethane/CNT composites as microwave absorbers are usually preferred in aeronautics applications. However, few studies have been conducted on polyurethane/CNT composites as microwave absorbers [12].

This paper describes the methods of synthesis used to produce MWCNT and studied the dielectric properties of the functionalized CNT with metal or metal alloy oxide nanomaterials embedded in a polyurethane matrix (PU) for the spectral range 5–50 GHz. Three sets of samples are developed in which each set comprises 5%, 10%, and 20% of magnetic nanomaterials like cobalt oxide (Co oxide), cobalt iron oxide (CoFe oxide), and iron oxide (Fe oxide). The morphological characteristics are also studied using TEM (transmission electron microscopy) and SEM (scanning electron microscope) images and finally the reflection loss calculation is carried out to examine the absorption efficiency of the samples with different thickness.

## 2. Materials and Methods

MWCNTs of inner diameter between 5 and 12 nm and outer diameter of 30–50 nm produced as described in our previous work [4] were used in the preparation of doped MWCNTs. The nanoparticles to decorate the surface of the MWCNTs were prepared using chemical co-precipitation technique. The cobalt-ferrite (CoFe_2_O_4_) were prepared by mixing 0.1 M of cobalt nitrate solution and 0.1 M of ferric nitrate solution in 200 mL of deionized water. The solution was stirred continuously during heating at 90 °C. 8 M of sodium hydroxide solution was applied as a reducing agent. It was applied dropwise to the heated solution. The formation of CoFe_2_O_4_ was observed with the changing of the solution color to brownish-black solution. Steering and heating at 90 °C was maintained for 90 min. Magnetic separation was used to isolate the nanoparticles, followed by washing several times to eliminate impurities. The formed particles were dried at 50 °C and stored for further use. The single atoms (Co or Fe) nanoparticles were prepared in the same procedure dissolving cobalt nitrate or iron nitrate in distilled water and utilizing sodium hydroxide as a precipitation agent.

The decoration of MWCNTs with metallic nanoparticles were performed by mixing the MWCNTs with the metallic nanoparticles for 12 h. The nanoparticles were first sonicated in distilled water for 5 min in ice bath. Due to the size of the particles, the solution demonstrated a colored suspension. Separately, the MWCNTs were also sonicated in distilled water for 5 min in ice bath. The MWCNTs, if left without mixing will settle at the bottom of the mixing tube. The two solutions were then mixed and stirred for 12 h. The nanoparticles were adsorbed on the MWCNTs surface. The mixture was allowed to settle for a few minutes. The change in color in supernatant from the nanoparticles color to clear solution indicates complete adsorption of the nanoparticles on the surface of the MWCNTs. The metal nanoparticles surface decorated MWCNTs were centrifuged at 3000 rpm, dried, and stored for further use.

**Figure 1:**
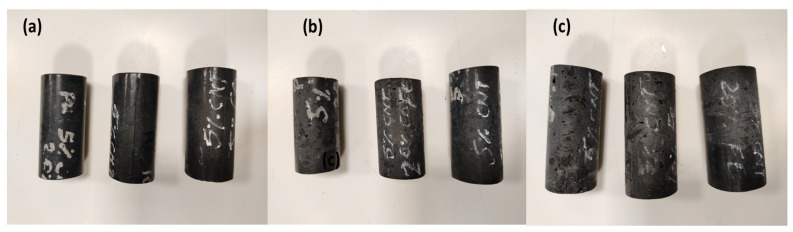
Functionalized samples (**a**) PU + 5%CNT + (5%, 10%, 20% Co), (**b**) PU + 5%CNT + (5%, 10%, 20% CoFe), (**c**) PU + 5%CNT + (5%, 10%, 20% Fe).

Functionalization consists of adding magnetic nanoparticles inside or outside the CNT. This process is usually done with a low content of nanoparticles. This condition is imposed such that the composite does not have a significant magnetic loss. The synthesis of functionalized CNT in polyurethane is carried out by adding 70 g of Reckli-FM-PU 48 polyurethane base (polyol mixture) to the needed quantity of metallic nanoparticles surface decorated MWCNTs; here 5 weight parentage of MWCNT was used. To ensure uniform distribution of the MWCNTs in the polyurethane base, the polyurethane-MWCNT mixture was heated (to reduce the viscosity of PU) via sonication for 15 min. The polyurethane base with MWCNT was allowed to cool down to room temperature after sonification, followed by the addition of 7 g of Reckli-FM-PU 48 hardener, and this mixture is poured into a hollow metal cylindrical mold. Then the mixture was kept for 24 h to dry and finally the composite was obtained with a height of 4 inches and a diameter of 1 inch as shown in Figure 1.

**Figure 2:**
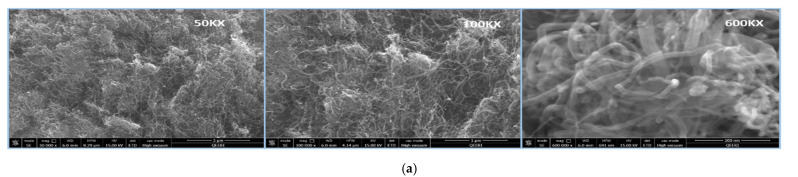

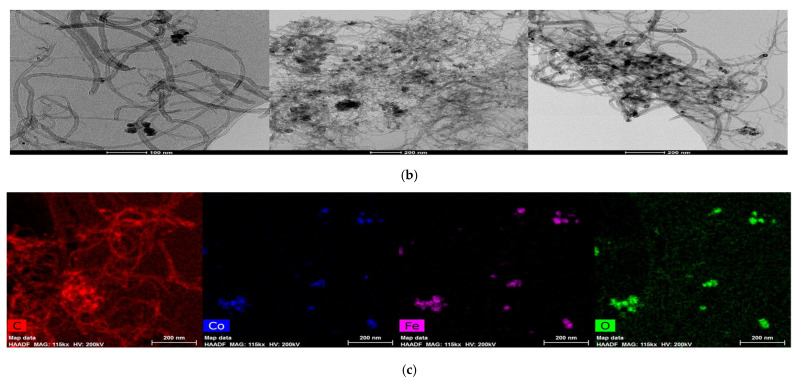
(**a**) SEM images for CNT with cobalt-iron oxide nanoparticles. (**b**) TEM images for CNT with cobalt-iron oxide nanoparticles. (**c**) Elemental mapping of cobalt iron oxide nanoparticles.

**Figure 3:**
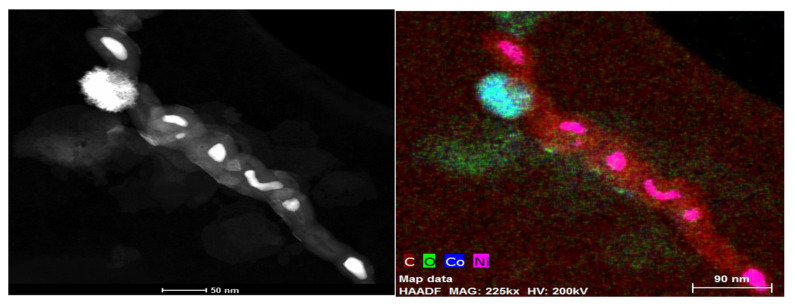
TEM of cobalt oxide decorating MWCNTs and elemental mapping.

**Figure 4:**
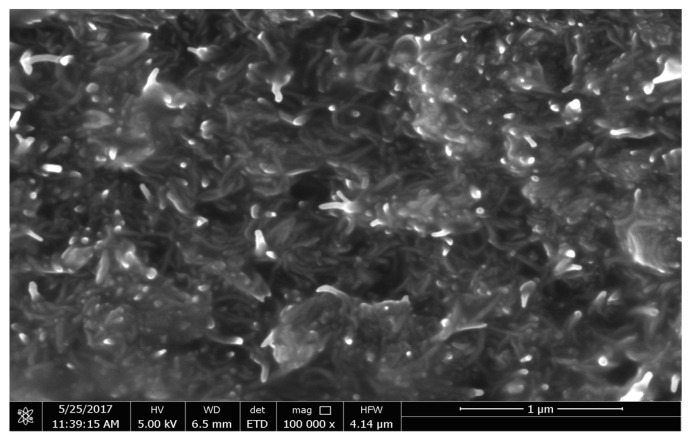
SEM of MWCNs impregnated in PU matrix.

The morphology of the nanoparticle-decorated MWCNTS is studied through the scanning electron microscope (SEM, JEOL 7800), transmission electron microscopy (TEM, FEI-Thermo scientific- TALOC C), and elemental mapping. Figure 2a shows the SEM image of cobalt iron oxide nanoparticles with different magnification of 50KX, 100KX, and 600KX. Figure 2b depicts the TEM image of the cobalt iron oxide nanoparticles with MWCNT, which reveals the morphology of nanoparticles attached to the surface of MWCNT. The images show spherical morphology of the nanoparticles with size ranging from 5 to 30 nm. The elemental mapping of the individual particles of C, Co, Fe, and O is shown in Figure 2c. TEM, SEM, and elemental mapping confirm the formation of crystalline cobalt iron oxide nanoparticles. Figure 3 depicts the TEM of cobalt nanoparticles decorating the surface of MWCNTs and elemental mapping. The morphology of the cobalt nanoparticles depicts a rod shape with a size of about 5 nm in length and 2 nm in diameter. The iron nanoparticles decorating the MWCNTs have a spherical morphology with average diameter of 5 nm. Figure 4 depicts the SEM image of PU impregnated with surface decorated MWCNTs. The image demonstrates a uniform distribution of the MWCNTs in the PU matrix.

## 3. Instrument Setup and Measurements

Different test methods used to predict the absorption properties can be obtained by the measurements of waveguide method [13], free space method [14], and coaxial line method [15,16,17]. This work used the open-ended coaxial probe technique. This method has the benefit of not having to rigidly fix the sample geometry to match the geometry of the sampling apparatus and the calibration process is simple and also the method can cover a wide range of frequency. However, this method of measurement will only provide the complex dielectric constant. Several researchers reported that the addition of magnetic nanoparticles like Co and Fe will not have significant effect on the complex permeability [3,18,19].

The system setup comprises a vector network analyzer, DAK (dielectric assessment kit) probe, Ethernet cable, and computer. This study used an Anritsu Vector Network Analyzer (VNA, MS4647B, ANRISTU), which can measure the S11 scattering parameter using the DAK probe connected to the VNA up to a frequency of 75 GHz. The probe DAK 1.2 developed by the SPEAG is used to measure the frequency-dependent dielectric constant and it can be used for a wide frequency range varying from 5–50 GHz. Ethernet cable is connected between the VNA and the computer. The DAK software is installed on the computer will extract the complex permittivity, permeability, tangent loss, conductivity parameters from the S_11_ measurements and this data can be used for the reflection loss calculations.

**Figure 5:**
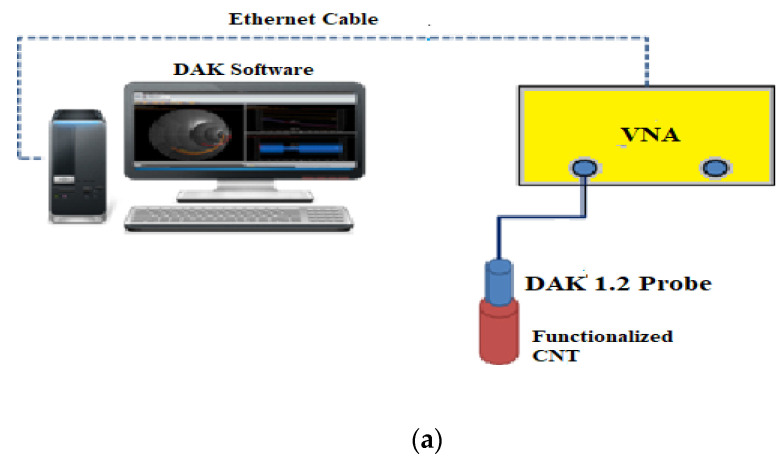

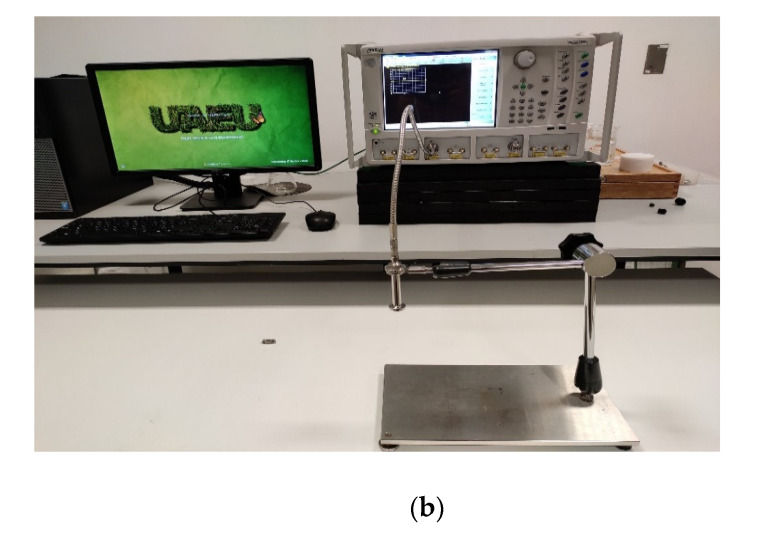
(**a**) Instrument setup for the measurement. (**b**) Measurement setup used in our lab.

The probe should be placed at an appropriate height and the sample being examined is brought directly into contact with the probe. Ultimate care is taken to avoid the movement of the probe and coaxial cable once it is set, as dielectric measurements are very sensitive to the reflected signal. The DAK has to be optimized for precise measurements, the calibration will normalize the magnitude and phase changes of the probe and coaxial cable. There are two methods of calibration available for the DAK system, Open, Short, and load (OSL) calibration and two loads (O-L_1_-L_2_) calibration. Open calibration is carried out by exposing the probe to the open air, for short calibration a copper strip is connected to the probe using a connecting block. Then the load calibration is usually done by the deionized water, its dielectric properties are known. While in the two-load method instead of a short calibration another load of known dielectric properties in the air is used. The effectiveness of calibration can be analyzed by the smith chart displayed in the DAK software. For accurate measurements the air gap between the measuring sample and the probe should be minimum. Figure 5 shows the measurement setup and Figure 5b depicts the measurement setup used in our lab.

**Figure 6:**
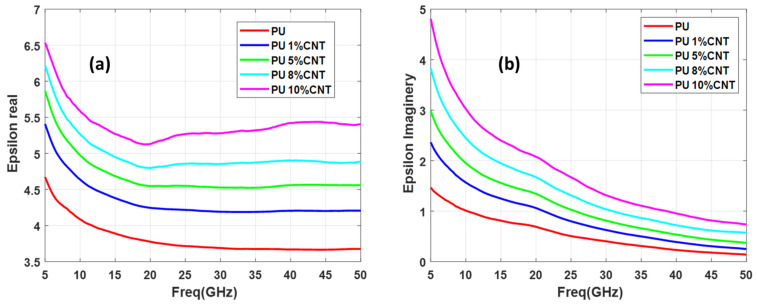
PU with different concentrations of CNT (**a**) epsilon real (**b**) epsilon imaginary.

For an efficient absorber, light weight is an important criterion especially for the stealth technology, thus the selection of CNT concentration is critical to maintain the sample weight. Figure 6 depicts the real and imaginary parts of the permittivity for the 0 wt.%, 5 wt.%, 8 wt.%, and 10 wt.% of CNT in the PU matrix. As the concentration of CNT increases, the real and imaginary of permittivity values increase too. A high value of 6.5 is obtained for the 10 wt.%CNT but moving toward the high frequency the real and imaginary parts of permittivity reduce to lower values, even near to zero for the imaginary values while the real part is almost constant in high frequencies. Considering the weight and the permittivity value, 5 wt.% was selected for the functionalization of CNT to obtain an efficient absorber.

**Figure 7:**
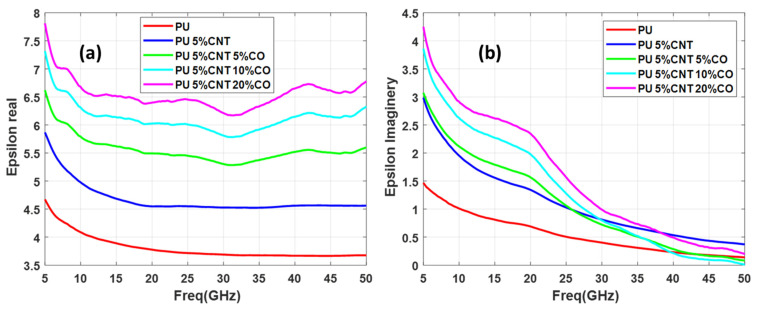
Permittivity of PU + 5%CNT with different concentrations of Co: (**a**) real part, (**b**) imaginary part.

**Figure 8:**
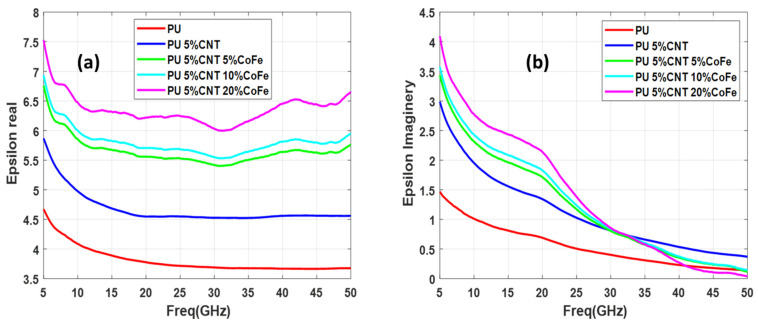
Permittivity of PU + 5%CNT with different concentrations of CoFe: (**a**) real part (**b**) imaginary part.

**Figure 9:**
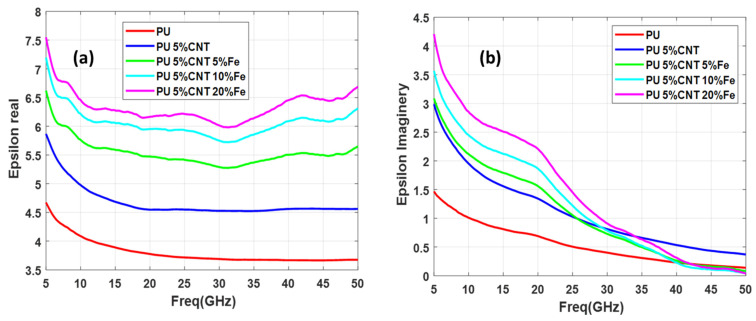
Permittivity of PU + 5%CNT with different concentrations of Fe: (**a**) real part (**b**) imaginary part.

In this study, three sets of samples of functionalized CNT are measured in each set having three samples of functionalization at different concentration levels. All the sample consists of 5 weight percentage (wt.%) of CNT and 5%, 10%, and 20% concentration of functionalizing metal nanoparticles (Co, Fe, and CoFe). Thus set 1 consists of PU + 5%CNT with 5%Co, PU + 5%CNT with 10%Co, and PU + 5%CNT with 20%Co, second set consists of PU + 5%CNT with 5%CoFe, PU + 5%CNT with 10%CoFe, and PU + 5%CNT with 20%CO, finally, the third set consists of PU + 5%CNT with 5%Fe, PU + 5%CNT with 10%Fe, and PU + 5%CNT with 20% Fe. The real and imaginary permittivity of the above-mentioned samples is illustrated in Figure 7, Figure 8 and Figure 9.

Relative complex permittivity and relative complex permeability are the two important parameters used to characterize the nanocomposites. The real part of permittivity measures the ability of the material to be polarized or energy stored and represents the potential for energy dissipation of the dielectric by which the energy derived from the electric field is transformed into thermal energy. Similarly, it measures the ability of a material to be magnetic field storage or magnetic loss respectively [4]. By analyzing Figure 7, Figure 8 and Figure 9 it is seen that the real part of the dielectric constant is increasing as the concentration of the functionalizing element (Co, CoFe, and Fe) is increased. Comparing these three samples it can be seen that CoFe and Fe follow the same pattern of the curves for both real and imaginary parts of the permittivity. The real part of permittivity has a high value in the lower frequencies, then it reduces and again around 32 GHz starts to increase but the imaginary part of the permittivity value is high at the lower frequency while in the higher frequency permittivity drops near to zero. The interaction of the electromagnetic energy with the material is highly related to the electrical properties of the material. Such interaction is related to material composition and content that can interact with the electric and magnetic forces produced by the stimulus electromagnetic fields. From an energy point of view, this interaction can be translated as energy storage, which describes energy dissipation (absorbed) inside the material; and energy storage, which relates to the lossless portion of energy exchange between the stimulating field and the material.

It is also noted that the curves exhibit a wavy shape or non-monotonous behavior, with several minima and maxima. Such a behavior has been previously reported for SWCNT/cross-linked polyurethane composites [12]. Complex permittivity properties of a material largely depend on contributions of various forms of polarizations such as interfacial, atomic, orientation, and electronic. Moreover, parameters such as percolation effect, CNT fiber aspect ratio, nano-particle size, and other elemental parameters highly influence the complex permittivity properties.

## 4. Reflection Loss Calculation

**Figure 10:**
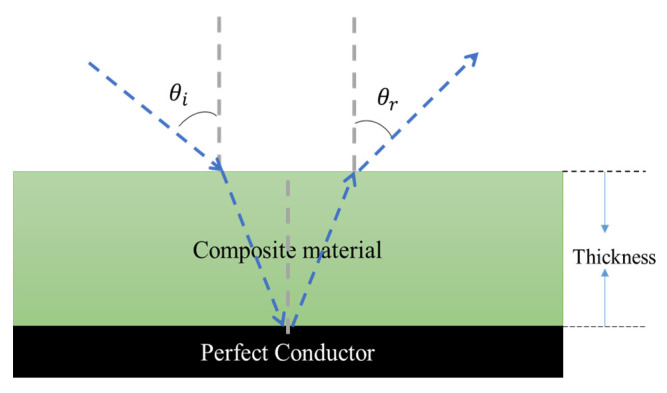
Schematic illustration of electromagnetic wave propagation through the composite material.

Reflection loss is the parameter used to determine the efficiency of the microwave absorption in the composite by using the complex permittivity values. Figure 10 illustrates the layered structure of the composite is back layered with a perfect conductor. When the electromagnetic wave incidents on the material it partially reflects at the air material interface, while another component penetrates through the barrier film and remains absorbed or dissipated within the material as thermal energy [20]. In this work the reflection loss of the single layer PU-CNT composite with functionalization is studied.

To reflect waves into the substrate, a metal surface is placed close to the substrate in order to maximize the absorption capacity. Transmitting waves are therefore negligible in microwave absorption with a metal background; hence, the reflection loss is measured as the difference between the initial incident wave and the final reflected wave [21]. The measurement of reflection loss is carried out with the formation of the S matrix [22], the layered structure can be represented by a 2 × 2 matrix whose coefficients are connected to the reflection and transmission coefficients. S, parameters specify the response of an N port network to a signal, there are four S parameters for a two-port network, namely S_11_, S_12_, S_21_, and S_22_, in which the first subscript denotes the input port and the second subscript is the output port [23]. This section discusses the reflection loss of the developed factionalized composites with a metal-backed perfect conductor at the normal incidence of the electromagnetic wave. The reflection measurement helps to design the exact composite material with the desired thickness and concentration to fulfill the requirements for the microwave application. The efficiency of the microwave absorption is related to the dielectric loss, thickness of the sample, and matching impedance. For nanocomposites, the reflection loss peak is moved to the lower frequency range by increasing the thickness of the sample [24] and also its parameter for tuning the absorption spectrum.

According to the theory of the transmission line, the reflection loss of electromagnetic radiation RL (dB) by normal incident wave at the surface of single-layer material backed by a perfect conductor [25] can be described as Equation (1).

(1) $$\mathrm{RL}\left(\mathrm{dB}\right)=20\;\mathrm{Log}\left|\frac{Z_{\mathrm{in}}+Z_{0}}{Z_{\mathrm{in}}-Z_{0}}\right|\;$$

(2) $$Z_{0}=\sqrt{\frac{\mu_{0}}{\varepsilon_{0}}}\;\approx 377\;\mathrm{ohm}\;$$

(3) $$Z_{\mathrm{in}}=Z_{0}\sqrt{\frac{\mu_{r}}{\varepsilon_{r}}}\tan h\left[j\left(\frac{\omega d}{c}\right)\sqrt{\mu_{r}\varepsilon_{r}}\right]$$

where $Z_{0}$ is the characteristic impedance of the free space, given by the Equation (2) and approximately equals to 377 ohms, $Z_{\mathrm{in}}$ is the input impedance of the metal-backed composite material and is obtained by the Equation (3) as [26] and depends on the composite electric and magnetic properties. Where $\mu_{r}$ is the relative complex permeability, $\varepsilon_{r}$ is the relative complex permittivity, (ω) as the frequency of the incident wave, d is the thickness of the absorbing material, and c is the speed of light in the free space. For an ideal microwave-absorbing material the impedance matching between free space and material-air interface should be attained that is $Z_{\mathrm{in}}=Z_{0}\;$ [27]. A good matching EM impedance condition can allow the incident microwave to have almost zero reflectivity. The attenuation factor $e^{-\alpha d}$, which works by lowering the wave amplitude in relation to the traveling distance d, controls the attenuation of electromagnetic energy inside the sample. The attenuation constant (α) is also influenced by the material’s electric and magnetic properties and defined by:

(4) $$\alpha =\omega {\left\{\frac{\mu \varepsilon }{2}{\left[1+{\left(\frac{\sigma }{\omega \varepsilon }\right)}^{2}\right]}^{1/2}-1\right\}}^{1/2}\;$$

**Figure 11:**
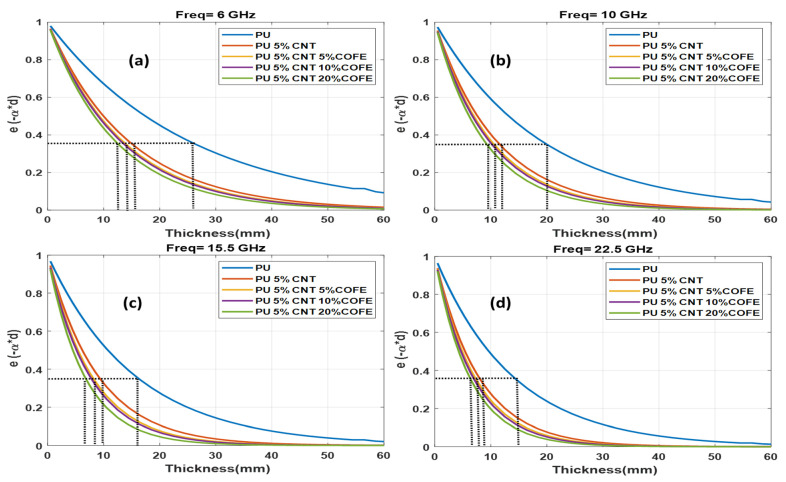
Comparison of attenuation factor for PU, PU 5%CNT, PU + 5%CNT + (5%, 10%, 20%) CoFe at different center frequencies (**a**) 6 GHz (**b**) 10 GHz (**c**) 15.5 GHz (**d**) 22.5 GHz.

Figure 11 depicts the attenuation factor at center frequencies of c band (6 GHz), X band(10 GHz), Ku band (15.5 GHz), and K band (22.5 GHz) against varying thickness for the samples of PU, PU 5%CNT, PU + 5%CNT + (5%,10%,20%) CoFe. As the frequency is increased, the curve moves toward the lower thickness and the wave amplitude decreases to a factor of $e^{-1}$ (about 37% of the original values) denoted by the dotted lines. From the sample, PU takes much thickness to attenuate the wave, as the concentration of the functionalizing element increases the attenuation distance decreases. The same trend is followed for the samples with Co and Fe functionalizing elements.

## 5. Results and Discussion

This section discusses the reflection loss of the nine samples developed with different concentrations (5%, 10%, and 20%) of the CNT composites with metal oxide (Co, CoFe, and Fe) functionalization. The reflection loss is calculated in the spectral range from 5–50 GHz and in all the samples there are several minima of reflection observed in the selected frequency range.

**Figure 12:**
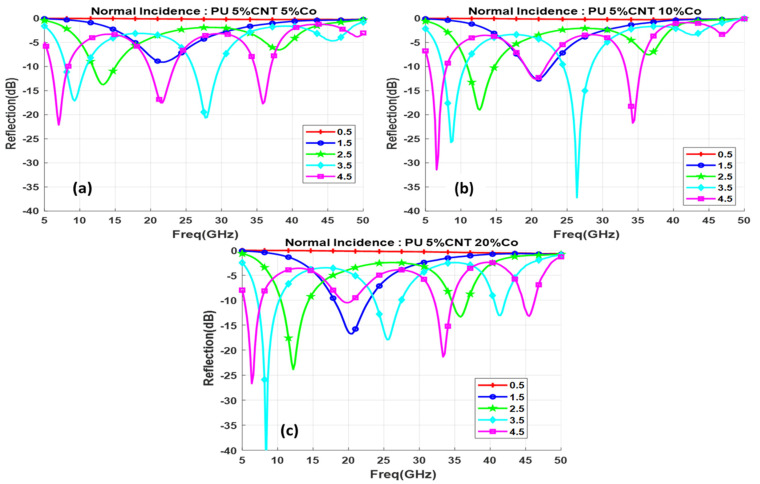
Reflection loss for functionalized composite with different thicknesses (d mm) for (**a**) 5% Co, (**b**) 10% Co, (**c**) 20% Co.

**Figure 13:**
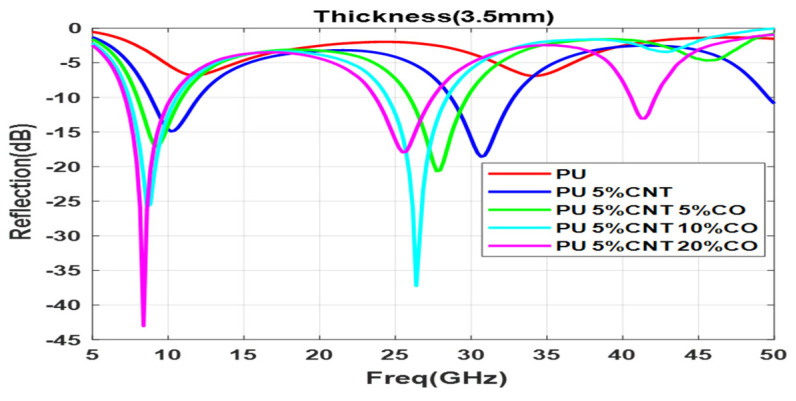
Reflection loss for different concentrations of Co at 3.5 mm thickness.

Functionalization of CNT with 5%, 10%, and 20% of Co nanomaterial is shown in Figure 12a–c with different thicknesses varying from (0.5–4.5 mm); as the concentration increases the composite shows better reflection values with small thickness, 5% Co composite does not have much effect on the reflection value with 1.5 mm but with the increase in concentration to 20% the reflection value reduced to −17 dB with 1.5 mm thickness. A minimum reflection of −43 dB is obtained for the composite with a 20% Co concentration with a thickness of 3.5 mm. By increasing the concentration of the functional elements, the minima moves to the lower frequency region due to the increase in the permittivity of the sample. 10 % Co composite with the thickness of 3.5 mm and 4.5 mm displays two prominent reflection loss minima below −30 dB. This implies most of the electromagnet waves get absorbed in the system. At 3.5 mm thickness, the reflection loss of −43 dB is obtained at 8 GHz frequency and shows an absorption bandwidth of 3.5 GHz, the high-frequency range has a minima at 26.5 GHz with a reflection loss of −37.5 dB as shown in Figure 13.

**Figure 14:**
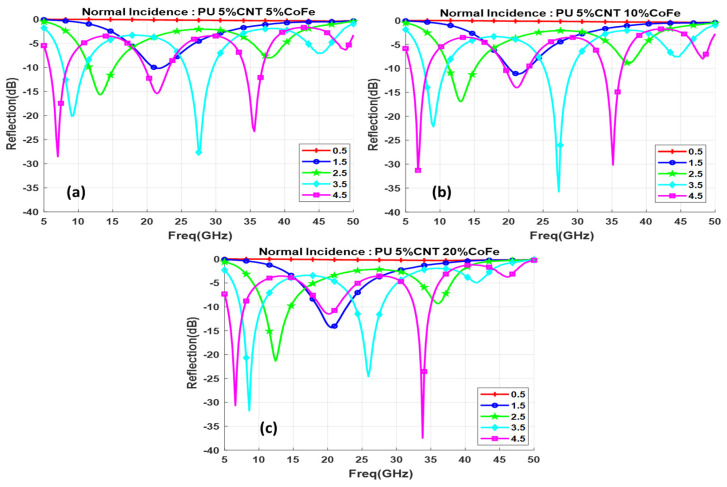
Reflection loss for functionalized composite with different thicknesses (d mm) for (**a**) 5% CoFe, (**b**) 10% CoFe, (**c**) 20% CoFe.

**Figure 15:**
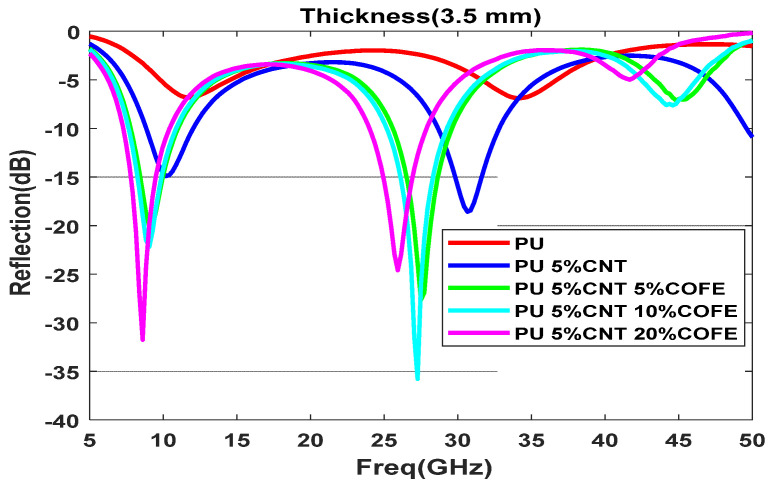
Reflection loss for different concentrations of CoFe at 3.5 mm thickness.

Figure 14a–c depicts the effect of the functionalized CNT composite with different concentrations of CoFe oxide nanoparticles on the reflection loss. 10% of CoFe concentration with a minimum value of −38 dB reflection loss is obtained at the frequency 34 GHz with a narrow bandwidth. Composite thickness varies from 2.5–4.5 mm shows minimal values of reflection at the frequency range 5–10 GHz. Comparing with Co functionalization, 5% CoFe has better reflection minimas with −28 dB is obtained with 3.5 mm and 4.5 mm thickness. The 10% and 20% CoFe are almost same as that of Co concentrations. Figure 15 shows the reflection loss for different concentrations of CoFe at 3.5 mm thickness. 4.5 GHz bandwidth is obtained for 5% and 10% CoFe in the frequency between 25 and 35 GHz.

**Figure 16:**
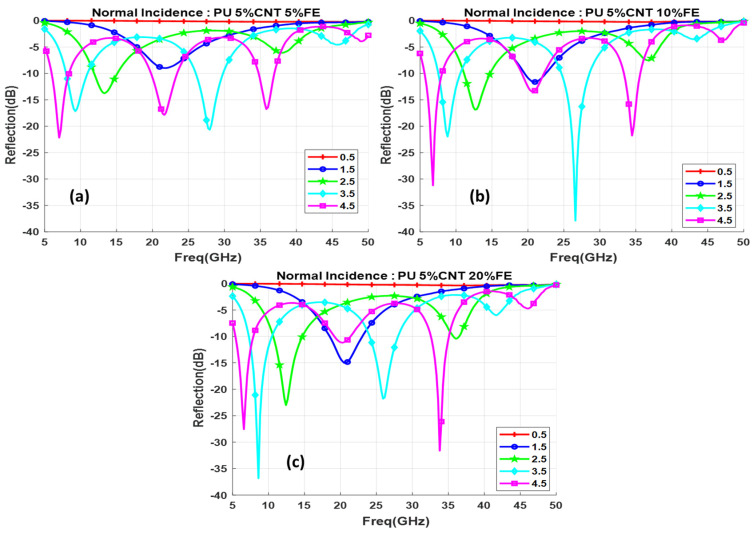
Reflection loss for functionalized composite with different thicknesses (d mm) for (**a**) 5% Fe, (**b**) 10% Fe, (**c**) 20% Fe.

**Figure 17:**
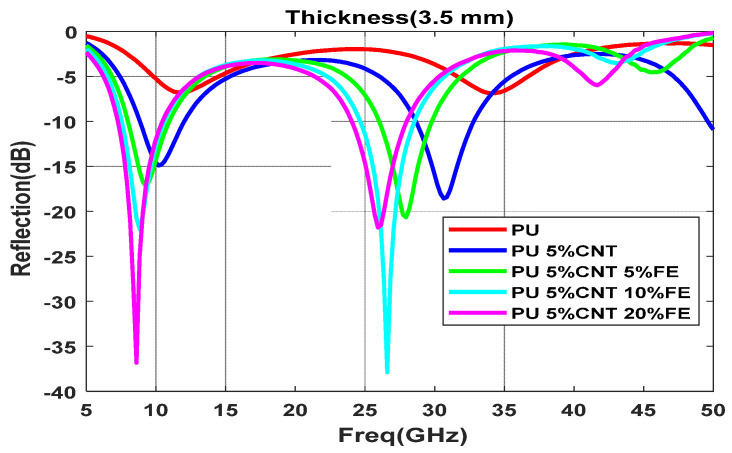
Reflection loss for different concentrations of Fe at 3.5 mm thickness.

Functionalization with Fe oxide nanoparticles obtained the minimum reflection loss of −37 dB for 10% and 20 % Fe concentrations. But the values are obtained in two different frequency ranges. 10% Fe has value in the higher frequency while for 20% the value obtained is in the lower frequency range as shows in Figure 16b but the bandwidth gets narrower, CoFe and Fe nanoparticle composed composites show almost similar behavior of curves. Figure 17 shows the reflection loss for different concentrations of Fe at 3.5 mm thickness.

## 6. Conclusions

Several concentrations of nanoparticles are used to make functionalized CNT composites: their dielectric properties are measured and compared with those of basic CNT composites in order to show the effect of functionalization on the electromagnetic properties and on the absorption efficiency of the CNT composites. Electrical properties were measured using the open-ended coaxial probes method. For a metal-backed single layer slab, the reflection loss characteristics for the samples were examined, and obtained a minimum reflection loss of −43 dB at 8 GHz with a thickness of 3.5 mm. 4.5 GHz bandwidth is obtained for 5% and 10% CoFe in the frequency between 25 and 35 GHz. This implies the functionalized CNT composites with the right composite thickness and concentration can be used to enhance the microwave absorption efficiency. Therefore, this composite will be a good option in light weight absorber with high absorption capability for the stealth applications.
